# Trueness and Precision of Eight Intraoral Scanners with Different Finishing Line Designs: A Comparative *In Vitro* Study

**DOI:** 10.1055/s-0042-1757568

**Published:** 2022-12-13

**Authors:** Mina Yahia Falih, Manhal A. Majeed

**Affiliations:** 1 Conservative Dentistry, College of Dentistry, Baghdad University, Iraq

**Keywords:** accuracy, intraoral scanner, finishing line, digital dentistry

## Abstract

**Objective** This study aimed to evaluate the accuracy in terms of trueness and precision of eight intraoral scanners (IOS) and the effect of different finishing line designs on the IOS's accuracy.

**Materials and Methods** Three printed models of the maxillary arch with maxillary right first molar virtually prepared with chamfer, shoulder, and vertical preparation designs were used as master models in this study. Each model was scanned 30 times with each IOS: Medit i700, Planscan Emerald S, CEREC Primescan, TRIOS 3, CS3600, MEDIT i500, Heron 3Disc, and Cerec Omnicam. The trueness was measured by superimposition of the scanned dataset made with IOS and the scanned dataset made with a lab scanner (In Lab Medit T710) that was used as a reference and the deviation was measured and expressed as a color-coded map by the metrology program (Medit compare, version 2.3.5.892), while precision was measured by the superimposition of the scans of each IOS on each other.

The data were analyzed statistically using repeated measure analysis of variance (ANOVA) test, one-way ANOVA test, and Bonferroni test at significance level of 0.05.

**Results** The tested IOS showed significant differences in trueness and precision. Medit i700 and CEREC Primescan recorded the highest precision with no significant difference between them, while Medit i700 recorded the highest trueness as compared to other IOS. Each IOS showed significant differences in trueness and precision with the three finishing line designs except CEREC Primescan and Heron 3 disc that showed no significant difference in trueness with the three finishing line designs and CS3600 that showed no significant difference in precision with the three finishing line designs.

**Conclusion** A significant difference in accuracy was found among the tested IOS and the type of finishing line design had a significant effect on IOS's accuracy.

## Introduction

Intraoral scanner (IOS) is a device that captures an optical impression of teeth and implants by means of light beam. Regardless of imaging technology type that is utilized by IOS, cameras necessitate the light to be projected. This beam of light is registered as images or video individually then estimate the points of interest and picked up by the software program. The primary two coordinates (x and y) of every point are assessed on the image, while the third coordinate (z) is counted depending on the “distance to the object technologies.” These technologies are supported by principles of optical-triangulation, active wave sampling, confocal microscopy, and stereo-photogrammetry. 1

The introduction of computer-aided design/computer-aided manufacturing technology in dentistry provided numerous advantages. 2 The most essential characteristic of IOS is that it has the opportunity to totally replace the standard impression, more comfortable for the patient as well as the elimination of impression material cost. Additional positive aspect is it allows direct evaluation of the impression. However, several limitations such as the struggle in detection of deep preparation margins, the occurrence of blood, and saliva prevent the acquisition of the scanned surface as well as the high cost of obtaining and maintaining the device. 3 IOS are presented for quite 30 years with the fast increase in the numbers of the available systems commercially. It seems to be a change in technology as several IOS have shifted from monochromatic image acquisition to systems of video-acquisition with color, whether with or without powdering. 4 5

Accuracy is measured in terms of trueness and precision (ISO 5725-1). 6 Trueness is the deviation between the scanned object with IOS from its true geometry (in this study the models scanned with in lab oral scanner). On the other hand, precision is the deviation between the repeated scans of the identical object executed with the identical IOS and with similar parameters. Three-dimensional (3D)-compare analysis is an approach that superimposes two surfaces after best-fit-alignment; this procedure is approved and utilized to measure the accuracy of IOS. 7

To assess the accuracy of IOS, the software creates a comparison between Standard Tessellation Language (STL) file of the IOS (target) and the reference scan's STL file using the best-fit algorithm function; this matching results as a linear deviations between the two scan sets that can be measured. 8 The discrepancies between the two scans (reference and target) are calculated by superimposition of the STL files derived from the two scans in dedicated software and using the function of best-fit algorithm. 9 It becomes essential to clarify that the linear distances must be used without their signs (absolute values) to prevent results canceling each other during the measurement of trueness and precision. 10

The IOS accuracy can be influenced by many variables such as ambient light, distance, substrate, preparation design, scanning strategy, practitioner experience, scanning technologies, hardware, and software version. 11 12

The IOS may be used clinically to scan preparations with different finishing line designs owing to different material requirements and clinician preference. Hence, this study was conducted to compare the accuracy of eight IOS in terms of trueness and precision when used to scan preparations with different finishing line designs.

## Materials and Methods

Three printed models of maxillary arch with maxillary first molar virtually prepared with different finishing line designs (chamfer, shoulder, and vertical preparation); these models were used in this study as master models as the 3D printed casts had a clinically adequate accuracy that can be used in diagnosis, fabrication of prosthetic restorations, and treatment planning, as it may be considered as a substitute for stone casts with. 13

A maxillary dentoform (Nissin, Kyoto, Japan) was scanned using Medit T710 (in lab scanner) and the maxillary right first molar of the dentoform received virtual preparation with the three different IOS for mentioned finishing line designs using 3D MAX designing program. 14

**Fig. 1:**
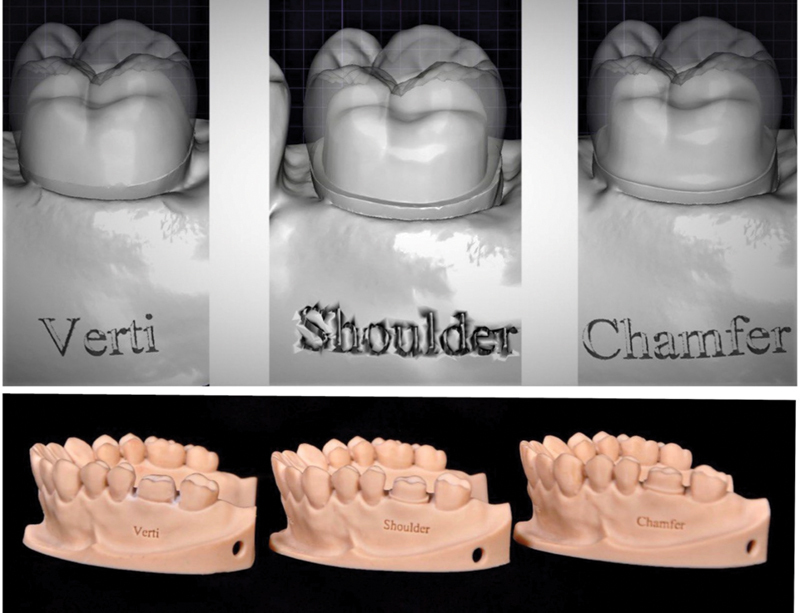
Virtual and printed models of the three finishing line design.

Each virtual model was then 3D printed using ASIGA MAX UV 3D ( Fig. 1 ). The models were then scanned with each one of the eight different IOS according to the following sample grouping: Group I: Medit i700 (Medit, Seoul, Korea), Group: II: Planscan Emerald S (Planmeca, Helsinki, Finland), Group III: CEREC Primescan (Dentsply-Sirona Dental Systems, Bensheim, Germany), Group IV: TRIOS (3Shape, Copenhagen, Denmark), Group V: CS3600 (Carestream, Dental Atlanta, GA, US), Group VI: Medit i500 (Medit, Seoul, Korea), Group VII: Heron 3Disc (Herndon, VA 20170, USA), and Group VIII: CEREC Omnicam (Dentsply-Sirona Dental Systems, Bensheim, Germany). Each group was subdivided into three subgroups according to the finishing line design as mentioned earlier. Each model was then scanned 30 times ( *n* = 30) with each IOS according to ISO 12836 (2015), which stated “in order to test the accuracy of a device in terms of trueness and precision, the measurement has to be repeated thirty times.” 15

Regardless of the type of IOS, all the digital scans were taken by the same operator to exclude the possible effect of variation in the operator's experience on the accuracy of IOS. 16

All the tested IOS and the reference scanner were calibrated at the time of installation using the respective calibration devices following the instructions of the manufacturer of each scanner. 17

The scanning procedure was performed according to the manufacturer's instructions of every IOS. For Medit i700, Medit i500, Heron, and TRIOS 3, OPB (occlusal, palatal, buccal) scanning procedure begun from the occlusal surface to the palatal surface then to the buccal surface, while for Planscan Emerald S, CEREC Primescan, and CS3600 OBP, scanning procedure begun from the occlusal surface to the buccal surface then to the palatal surface. For CEREC Omnicam, the scanning procedure begun from the occlusal, the buccal then the palatal surface of posterior teeth, then tipping movement on the anterior teeth, followed by the occlusal, the buccal, and the palatal surface of the posterior teeth of the opposite side.

The accuracy measurement was done based on ISO 5725 in which a combination of trueness and precision was approved to define the accuracy of the measurement method. 6 For the measurement of the trueness, the scanned datasets made with IOS were overlapped on the scanned dataset made with in lab scanner (Medit T710). The STL files were imported to a metrology program (Medit compare, version 2.3.5.892), where IOS meshes and in lab scanner meshes were superimposed and therefore the deviations measured as root mean square (RMS) and expressed as a color-coded map. For the measurement of precision, the scanned datasets of every IOS were overlapped on one another and also the deviations were measured as RMS and expressed as color-coded map same as trueness.

Many studies used laboratory scanners to measure the IOS's trueness to create a standard reference model as it is considered one of the standard methods for measuring. 9 18

Medit T710 (Medit T710, Seoul, South Korea) a standard laboratory scanner was used as a reference scanner for the measurement of the trueness of various IOS because it is the most up-to-date version of in lab scanner introduced by Medit Company. It works with blue-light scanning technology and incorporates a four 5.0 MP camera system, 19 ensuring a significant accuracy (<4µm) according to (ISO-12836). 15

**Fig. 2:**
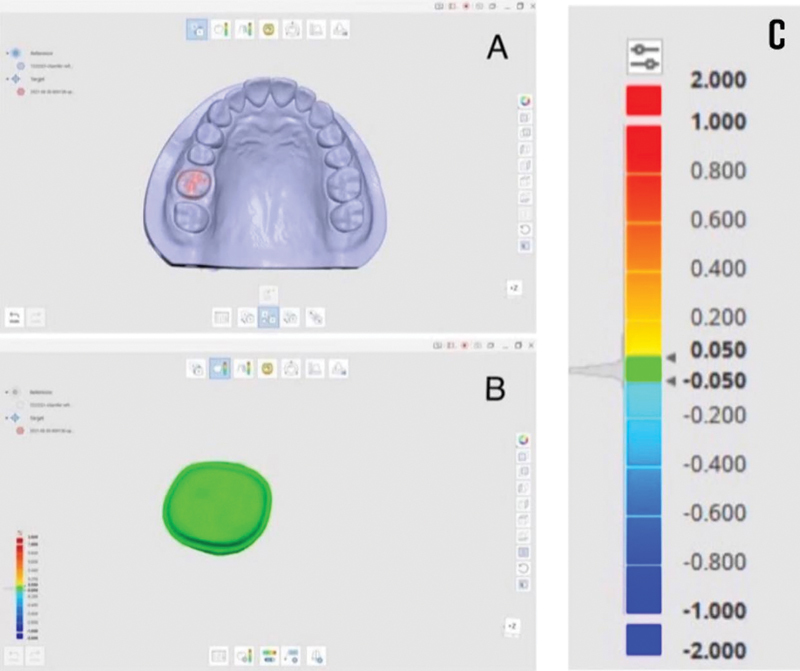
( **A** ) Best-fit alignment, ( **B** ) superimposition of reference, and target data ( **C** ) color-coded map for the deviation between the reference and measured data.

The scanned data were compared employing a 3D inspection software program (Medit compare, version 2.3.5.892.) (Seoul, South Korea) that is a metrology program used to analyze and compare 3D meshes. The information for both references and targets (the scanned data to be compared) was uploaded into the program and the area of interest of 3D meshes was isolated as the isolation procedure was done inside the software, regarding the margins of the preparation and the boundary meshes to be compared with the reference data. For every finishing line design, the information of the tested groups from (TG 1 to TG 30) was imported into Medit compare within the target section and also the reference data were imported to the reference section. The system automatically aligned the reference and therefore the target. Then will apply the “initial alignment” ( Fig. 2A ). The function of the software was performed to superimpose the IOS meshes over the reference mesh then it will be followed by the function of the “best-fit alignment” that focuses on the alignment of the isolated area of interest to make sure the overlapping of the two meshes will be done precisely. In order to analyze the deviation that occurred between the reference and target data, the “3D Compare” function was employed by projecting all paired points with a color-coded map created to display the deviation patterns of the investigated surfaces ( Fig. 2B ).

The color-coded map was established to indicate deviations between ± 50 µm within the color-coded map scale, the displacement that directed outward will be appeared toward the red spectrum while displacement that directed inward will be appeared toward the blue spectrum. The areas with no deviation are being appeared in green spectrum that means the deviation is less than ± 1 µm between the compared surfaces. The whole procedure was performed for all IOS meshes so as to get numerical values (RMS) for every design.

To achieve the precision values, each intraoral scanner mesh was compared with all the opposite meshes of every design within its group (calculated by the intragroup overlapping) following the identical protocol ( Fig. 2C ).

Statistical analysis was performed by using Statistical Package for Social Science (SPSS version 25). Data were considered normally distributed according to the central limit theorem as the sample size in each subgroup was 30. 20

Repeated measure analysis of variance (ANOVA) test was used to look for the significance of the mean difference of trueness and precision among the different groups with each finishing line design at a level of significance of 0.05. Bonferroni test was used for multiple comparisons among the groups at a level of significance of 0.05. One-way ANOVA test was used for comparison of the trueness and precision of each IOS with different finishing line designs at a level of significance of 0.05.

## Results

The descriptive statistics including the mean deviation (in µm), standard deviation, and minimum and maximum values of the trueness and precision of the eight IOS for the three preparation designs are shown in Table 1 . For trueness, the least mean deviation (i.e., the highest trueness) was recorded by Group I (Medit i700), while the highest mean deviation (i.e., the lowest trueness) was recorded by Group V CS3600 for chamfer and shoulder finishing line design and by Group VIII (CEREC Omnicam) for vertical preparation.

**Table 1:** Descriptive statistics of the trueness and precision

Trueness		Finishing line designs											
Group	N	Chamfer finishing line				Shoulder finishing line				Vertical preparation			
		Mean	SD	Min.	Max.	Mean	SD	Min.	Max.	Mean	SD	Min.	Max.
I	30	0.012	0.001	0.011	0.014	0.016	0.001	0.015	0.017	0.015	0.002	0.012	0.019
II	30	0.027	0.002	0.022	0.030	0.033	0.004	0.029	0.041	0.029	0.004	0.021	0.038
III	30	0.022	0.002	0.019	0.028	0.030	0.002	0.028	0.036	0.025	0.029	0.014	0.170
IV	30	0.025	0.006	0.018	0.044	0.027	0.004	0.022	0.038	0.018	0.005	0.014	0.036
V	30	0.028	0.008	0.020	0.049	0.038	0.006	0.026	0.051	0.029	0.014	0.012	0.074
VI	30	0.014	0.002	0.011	0.024	0.019	0.001	0.017	0.023	0.025	0.030	0.015	0.180
VII	30	0.021	0.013	0.014	0.080	0.024	0.006	0.017	0.052	0.027	0.009	0.017	0.063
VIII	30	0.023	0.001	0.020	0.025	0.029	0.003	0.026	0.043	0.031	0.007	0.027	0.056
**Precision**		**Chamfer finishing line**				**Shoulder finishing line**				**Vertical preparation**			
**Group**	***n***	**Mean**	**SD**	**Min**	**Max**	**Mean**	**SD**	**Min**	**Max**	**Mean**	**SD**	**Min**	**Max**
I	30	0.009	0.002	0.005	0.014	0.006	0.001	0.005	0.009	0.006	0.001	0.005	0.008
II	30	0.015	0.002	0.010	0.021	0.018	0.005	0.013	0.028	0.014	0.004	0.009	0.026
III	30	0.007	0.003	0.005	0.021	0.007	0.003	0.005	0.023	0.005	0.001	0.004	0.007
IV	30	0.020	0.010	0.007	0.045	0.021	0.015	0.008	0.054	0.011	0.009	0.007	0.042
V	30	0.024	0.016	0.012	0.099	0.022	0.010	0.011	0.060	0.030	0.017	0.012	0.078
VI	30	0.010	0.003	0.007	0.020	0.010	0.002	0.007	0.017	0.014	0.005	0.011	0.035
VII	30	0.020	0.002	0.015	0.025	0.016	0.002	0.013	0.023	0.018	0.007	0.010	0.034
VIII	30	0.012	0.004	0.008	0.027	0.010	0.002	0.007	0.017	0.017	0.009	0.009	0.051

Abbreviation: SD, standard deviation.

On the other hand, for the precision, the lowest mean deviation (i.e., the highest precision) was recorded by Group I Medit i700 and Group III (Cerec Primescan) for all the preparation designs with no significant difference between both types of IOS, while the highest mean deviation (i.e., the lowest precision) was recorded by group V (CS3600) IOS with all types of finishing line designs ( Table 1 ).

The comparison of significance was done by repeated measure ANOVA test and by one-way ANOVA test designs at a level of significance of 0.05 ( Tables 2 and 4 ). For each finishing line design, the multiple comparisons of the trueness and precision among different IOS were done by Bonferroni test results that revealed statistically significant differences among the groups ( Table 3 ).

**Table 2:** Repeated measure ANOVA for the comparison of the trueness and precision of the eight IOS of the different finishing line design

Preparation Designs	Trueness					Precision				
	Type III sum of squares	df	Mean square	*f* -test	*p* -Value	Type III sum of squares	df	Mean square	*f* -test	*p-* Value
Chamfer F.L	0.007	2.200	0.003	27.617	0.000	0.008	1.975	0.004	20.974	0.000
Shoulder F.L	0.011	3.437	0.003	100.063	0.000	0.008	2.203	0.004	24.641	0.000
Vertical prep.	0.007	2.602	0.003	3.713	0.019	0.013	2.640	0.005	26.589	0.000

Abbreviations: ANOVA, analysis of variance; IOS, intraoral scanners.

**Table 3:** Bonferroni test for multiple comparisons of the trueness and precision of the eight IOS of the different finishing line design

Groups		Bonferroni test for trueness			Bonferroni test for precision		
		Chamfer	Shoulder	Vertical	Chamfer	Shoulder	Vertical
		*p* -Value	*p* -Value	*p* -Value	*p* -Value	*p* -Value	*p* -Value
I	II	0.000	0.000	0.000	0.000	0.000	0.000
	III	0.000	0.000	1.000	0.735	1.000	0.571
	IV	0.000	0.000	0.095	0.000	0.000	0.057
	V	0.000	0.000	0.000	0.000	0.000	0.000
	VI	0.465	0.000	1.000	0.432	0.000	0.000
	VII	0.028	0.000	0.000	0.000	0.000	0.000
	VIII	0.000	0.000	0.000	0.003	0.000	0.000
II	III	0.000	0.008	1.000	0.000	0.000	0.000
	IV	1.000	0.000	0.000	0.128	1.000	1.000
	V	1.000	0.003	1.000	0.153	1.000	0.002
	VI	0.000	0.000	1.000	0.000	0.000	1.000
	VII	0.534	0.000	1.000	0.000	1.000	0.197
	VIII	0.000	0.006	1.000	0.273	0.000	1.000
III	IV	0.488	0.011	1.000	0.000	0.001	0.026
	V	0.006	0.000	1.000	0.000	0.000	0.000
	VI	0.000	0.000	1.000	0.009	0.026	0.000
	VII	1.000	0.000	1.000	0.000	0.000	0.000
	VIII	0.030	1.000	1.000	0.000	0.022	0.000
IV	V	0.836	0.000	0.005	1.000	1.000	0.000
	VI	0.000	0.000	1.000	0.000	0.009	1.000
	VII	1.000	0.917	0.001	1.000	1.000	0.214
	VIII	1.000	0.903	0.000	0.018	0.010	0.650
V	VI	0.000	0.000	1.000	0.003	0.000	0.001
	VII	0.813	0.000	1.000	1.000	0.171	0.083
	VIII	0.049	0.000	1.000	0.026	0.000	0.011
VI	VII	0.089	0.007	1.000	0.000	0.000	1.000
	VIII	0.000	0.000	1.000	1.000	1.000	1.000
VII	VIII	1.000	0.009	1.000	0.000	0.000	1.000

Abbreviation: IOS, intraoral scanners.

**Table 4:** One-way ANOVA test comparing trueness and precision of each IOS with different finishing line design

	Trueness						Precision					
Groups		Sum of squares	df	Mean square	*f* -test	*p* -Value		Sum of squares	df	Mean square	*f* -test	*p* -Value
I	Between groups	0.000	2	0.000	82.930	0.000	Between groups	0.000	2	0.000	40.836	0.000
	Within groups	0.000	87	0.000			Within groups	0.000	87	0.000		
	Total	0.000	89				Total	0.000	89			
II	Between groups	0.000	2	0.000	21.178	0.000	Between groups	0.000	2	0.000	11.041	0.000
	Within groups	0.001	87	0.000			Within groups	0.001	87	0.000		
	Total	0.001	89				Total	0.002	89			
III	Between groups	0.001	2	0.001	1.970	0.146	Between groups	0.000	2	0.000	4.826	0.010
	Within groups	0.025	87	0.000			Within groups	0.001	87	0.000		
	Total	0.026	89				Total	0.001	89			
IV	Between groups	0.001	2	0.001	26.369	0.000	Between groups	0.002	2	0.001	6.731	0.002
	Within groups	0.002	87	0.000			Within groups	0.011	87	0.000		
	Total	0.004	89				Total	0.013	89			
V	Between groups	0.002	2	0.001	8.736	0.000	Between groups	0.001	2	0.001	2.566	0.083
	Within groups	0.008	87	0.000			Within groups	0.020	87	0.000		
	Total	0.010	89				Total	0.021	89			
VI	Between groups	0.002	2	0.001	3.237	0.044	Between groups	0.000	2	0.000	15.996	0.000
	Within groups	0.026	87	0.000			Within groups	0.001	87	0.000		
	Total	0.028	89				Total	0.001	89			
VII	Between groups	0.001	2	0.000	2.643	0.077	Between groups	0.000	2	0.000	3.705	0.029
	Within groups	0.008	87	0.000			Within groups	0.002	87	0.000		
	Total	0.009	89				Total	0.002	89			
VIII	Between groups	0.001	2	0.000	11.252	0.000	Between groups	0.001	2	0.000	11.252	0.000
	Within groups	0.002	87	0.000			Within groups	0.003	87	0.000		
	Total	0.003	89				Total	0.004	89			

Abbreviations: ANOVA, analysis of variance; IOS, intraoral scanners.

## Discussion

It is worth mentioning that there is no clear cut that is more important, trueness or precision, for determining the accuracy of IOS. However, up to the researchers' knowledge, it seems that the trueness is more important than precision as it reflects how far the scanned data meet reality, while precision is more dependent on the operator's skill and handling of the device. The results of this study showed a significant difference in trueness and precision between the different IOS. It is worth to point that no single factor could be considered responsible for these differences. The tested IOS differ in their optical scanning technology, scanning strategy, and capture principles. 21

The superiority of trueness and precision of Medit i700 may be mainly associated with its scanning technology that allows an AWS module and one camera only. This module is designed as an off-axis aperture that relies on a circular path during its movement around the optical axis, a rotation of the target points on a circle on the image plane will be produced in response to this movement and also the target point's depth data probably came from the radius of the circular point pattern produced by each and advocated any system with a camera to function in 3D; therefore, it minimizes the need for multiple cameras to accumulate 3D geometries. 22

The most beneficial characteristic of this technology is that in order to capture depth information it necessitates only one optical path. 23

The superiority of the accuracy of IOS based on AWS technology is well-established by Kachhara et al who made a systematic review, which assessed eight *in vitro* studies. These studies compared the deviation that appeared in length and angle between the implant scan bodies of the obtained STL files from the scanned data to the real values of the master model obtained by an industrial 3D coordinated measurement machine. The studies showed that AWS technology achieves the smallest amount of error rate. 1 This study agreed with Kim et al who evaluated the accuracy of nine IOS with different scanning technologies for complete arch scanning and located a major effect of scanning technology on the accuracy of IOS that showed the AWS technique provided the best results in trueness. 24 On the contrary, this finding disagrees with Park et al whose results were inconclusive regarding which technology is the best to achieve an accurate scanning. 25

In this study in spite of the similarity in scanning technology of Medit i700 and Medit i500, Medit i700 showed more accuracy, which may be due to the improvement in the scanner wand itself including an increased scanning frame per second FPS up to 70 for Medit i700 compared to 30 FPS for Medit i500. Moreover, Medit i700 has a smaller tip size (22.2mm, 15.9mm) and lighter weight (245 g) than Medit i500 that has a larger tip size (18 mm, 15.2mm) and heavier weight (276g), 19 which may ease the handling of the device and improve its accuracy. 25

The results of this study showed that CEREC Primescan had comparable precision to Medit i700 despite the dissimilarity in their scanning technology. This might be attributed to the innovative Smart Pixel Sensor that may be a property found only during this scanner process, even at a measuring depth up to 20 mm dynamic lens that completes for each second approximately 10 movements by permitting the “Dynamic Depth Scan.” In addition to the intelligent processing, which has the ability to filter, process, and compress the high-volume data that enables the calculation of the models to be faster and has an idealistic sharpness and high precision. 26

In addition, the CEREC has the ability to activate an automatic shake detection system, which allows the images to be assimilated on condition that the camera is totally still. 27 On the other hand, the lower trueness recorded by CEREC Primescan, CEREC Omnican, and CS3600 can be associated with these IOS that are based on triangulation scanning technology, within which errors could occur during the estimation of “the distance to the object” that is contrariwise proportional to the distance between the light and the position of the detector, and therefore this prohibits the distance between the effective position of the lens and the detector position to be made large. 22

Meanwhile, Trios and Planmeca Emerald are based on confocal laser scanner microscopy technology. The uniqueness of this method is that it has the ability to get different position of the focal plane without the need to move the scanner in relevant to the scanned object; however, the time for the 3D surface acquisition has to be adequately short in order to reduce the bias that could occur during unintentional relative movements between the probe and the teeth. 22

In the meantime, Heron 3Disc IOS is based on the active stereo-imaging scanning technology; however, the information related to this technology is limited. The second possible cause for the variations in the accuracy of the tested IOS could be related to the differences in the scanning strategy of each IOS. The scan path means that IOS should be used in keeping with an exact movement to improve the accuracy of the model. 24

Many scientific studies showed the effective role of a scanning protocol in order to have accurate scans. 21 However, during this study each IOS was used following the manufacturer's recommendations. When a scan of the whole arcade is required, many strategies are described by manufacturers. One among these strategies was based on a linear movement started on all occlusal-palatal surfaces followed by buccal surface (OPB). This strategy was used for Medit i700, Medit i500, Trios, and Heron 3DISC in keeping with their manufacturers' recommendation. Richert et al suggested that this strategy has the ability to limit locative distortion as it finishes the capturing procedure at the initial position, avoiding an overall one-way error. 28

The superiority of OPB strategy was agreed with Müller et al and Stefanelli et al, who found that OPB is more accurate in terms of trueness and precision and that they suggested the cause is because of that it scans the surfaces without interruption, and hence introduces fewer distortion. 9 29 However, Oh et al suggested that there was no significant effect of the scanning strategy on the precision of IOS. 10

The third possible cause for the variations in the accuracy of the tested IOS may be the acquisition method or so-called “data capturing mode” of IOS. According to Nedelcu and Persson, IOS can be categorized into image-acquisition and video-acquisition based on the image recombination of the IOS. 30

Regardless of the scanning technology and scanning strategy, it has been suggested that the data capturing mode could have an effective role on the scan accuracy. IOS with a continual scanning flow (video) have shown higher accuracy than photo-based IOS. 31

During this study, all the tested IOS work with video-based acquisition methods except CS3600 and Heron 3Disc that were image-based. Kim et al compared the accuracy of nine IOS with different data capture modes CS3500, E4D, FastScan, and iTero by individual images versus CEREC Omnicam, PlanScan, Trios, True Definition, and Zfx IntraScan by video sequence. They found that video-based acquisition method trueness median was 56.45 and 70.55 µm for photo-based acquisition method. 24 However, Park showed no significant effect of the acquisition method, whether video-based or photo-based, on the accuracy of the IOS. 32

## Conclusions

Within the limitations of this *in vitro* study, the subsequent conclusions could be considered:

- There was a significant difference in trueness and precision of the eight IOS tested in this study. Medit i700 recorded the highest trueness, while the highest precision was recorded by Medit i700 and CEREC Primescan.
- There was a significant effect of the finishing line design on the accuracy of IOS. In general, regardless the type of IOS, the chamfer finishing line design provided the highest trueness, while the vertical preparation design provided the highest precision.
